# The Role of Timely Angiography in Elderly Patients Presenting With Lower Gastrointestinal Bleeding

**DOI:** 10.7759/cureus.47701

**Published:** 2023-10-26

**Authors:** Haider Ghazanfar, Nismat Javed, Bhavna Balar

**Affiliations:** 1 Gastroenterology, BronxCare Health System, Bronx, USA; 2 Internal Medicine, BronxCare Health System, Bronx, USA

**Keywords:** management, embolization, angiography, lower gi bleed, lower gastrointestinal tract

## Abstract

Lower gastrointestinal bleeding (LGIB) is associated with significant morbidity and mortality in the elderly population. Timely diagnosis and establishing the etiology of the LGIB can guide appropriate treatment and management. Our patient is a 91-year-old female who presented to the ER with the complaint of several episodes of hematochezia that started four hours before her presentation. The patient underwent an urgent CT angiography showing active bleeding in the proximal ascending colon. She underwent a super-selective arteriogram followed by embolization of the ascending colon arterial culprit bleeding territory using two coils. Her clinical condition improved, and she had no further episodes of hematochezia. Her case highlights the importance of timely diagnosis of the underlying etiology of a patient presenting with LGIB.

## Introduction

Lower gastrointestinal bleeding (LGIB) has an estimated annual incidence of hospitalization of approximately 36/100,000 population [1]. The hospitalization rates are even higher in the elderly [1]. The most common causes of LGIB include diverticulosis, colorectal polyps, and hemorrhoids [2]. In the United States, the prevalence of admissions due to LGIB has increased with time. The clinical presentation of patients varies from self-limited painless hematochezia to hemodynamic instability [1]. It is, therefore, important to diagnose the underlying etiology of LGIB bleeding using both clinical and imaging criteria. In this regard, CT angiography plays an important role [3]. Here, we present the case of a 91-year-old female who presented with LGIB, successfully treated with timely angiography and embolization of the bleeding artery.

## Case presentation

This is a case of a 91-year-old female who was brought to the ER because of three episodes of a large amount of painless rectal bleeding that started four hours before presentation. She has a history of chronic constipation and usually has bowel movements once in three days. The patient denied nausea, vomiting, hematemesis, diarrhea, or melena. Her last colonoscopy, done three years ago for evaluation of hematochezia, had shown extensive diverticulosis and hemorrhoids. The patient's medical history was significant for hypertension and a stroke with left-sided weakness. Her past surgical history was significant for hysterectomy.

Upon presentation to the ER, her blood pressure was 158/101 mmHg, heart rate was 89 beats/minute, respiration rate was 18/minute, and temperature 98.10F. Her initial physical examination revealed a soft, non-tender abdomen. The rest of the physical examination was unremarkable except for bright red blood on the rectal examination. The patient's initial laboratory workup is mentioned in Table 1.

**Table 1:** Laboratory investigations BUN: blood urea nitrogen, APTT: partial thromboplastin time, PT: prothrombin time, INR: international normalized ratio

Investigation	Value
Complete blood count	
White blood cell	9.8 (4.8-10.8 k/ul)
Hemoglobin	11.5 (12.0-16.0 g/dl)
Hematocrit	34.9 (42.0-51.0%)
Platelet	209 (150-440 k/ul)
Electrolytes	
Sodium	140 (135-145 mEq/L)
Potassium	4.2 (3.5-50 mEq/L)
Bicarbonate	23 (24-30 mEq/L)
Chloride	107 (98-108 mEq/L)
Glucose	121 (70-120 mg/dL)
BUN	14.0 (70-120 mg/dL)
Creatinine	0.8 (8-26 mg/dL)
Coagulation profile	
APTT	31.5 (27.2-39.6)
PT	12.2 (9.9-13.3)
INR	1.04 (0.85-1.14)

**Figure 1:**
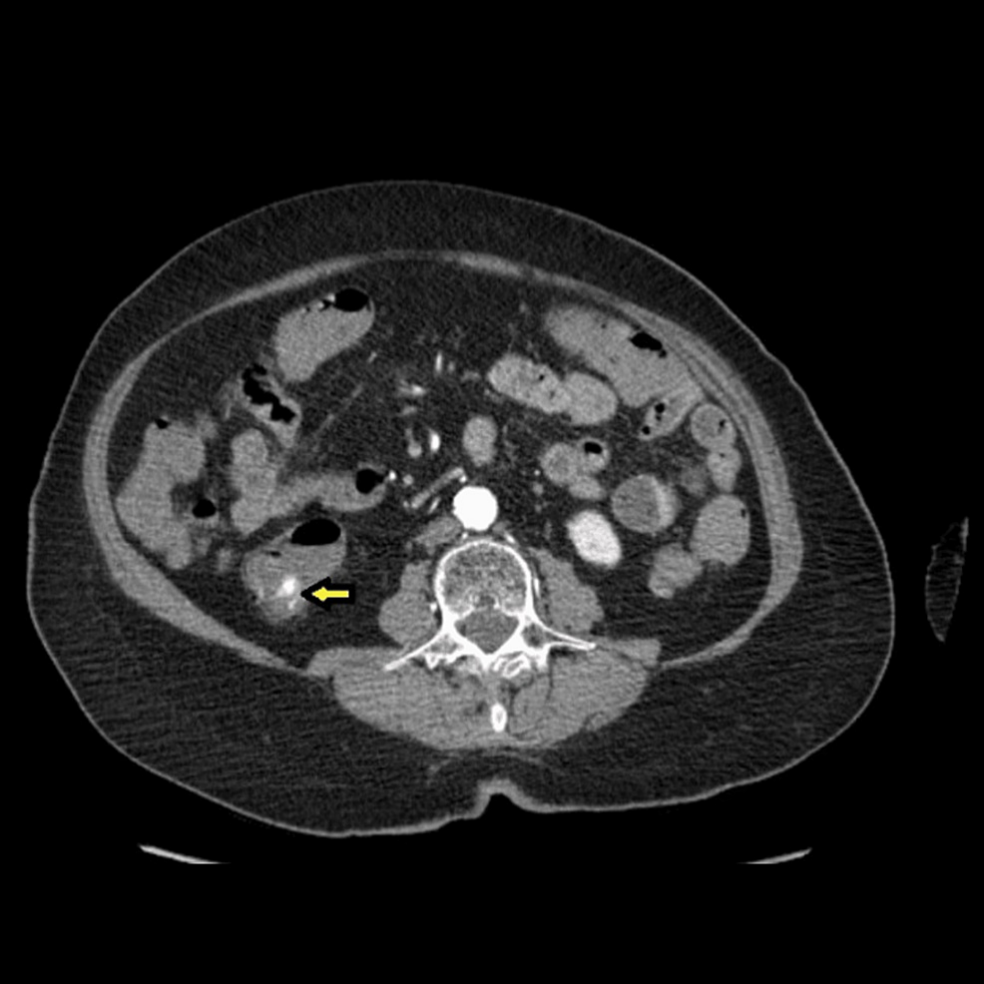
CT of the abdomen showing active bleeding in the proximal ascending colon (yellow arrow)

The patient underwent an urgent CT angiography of the abdomen and pelvis, which showed active bleeding in the proximal ascending colon (Figure 1).

**Figure 2:**
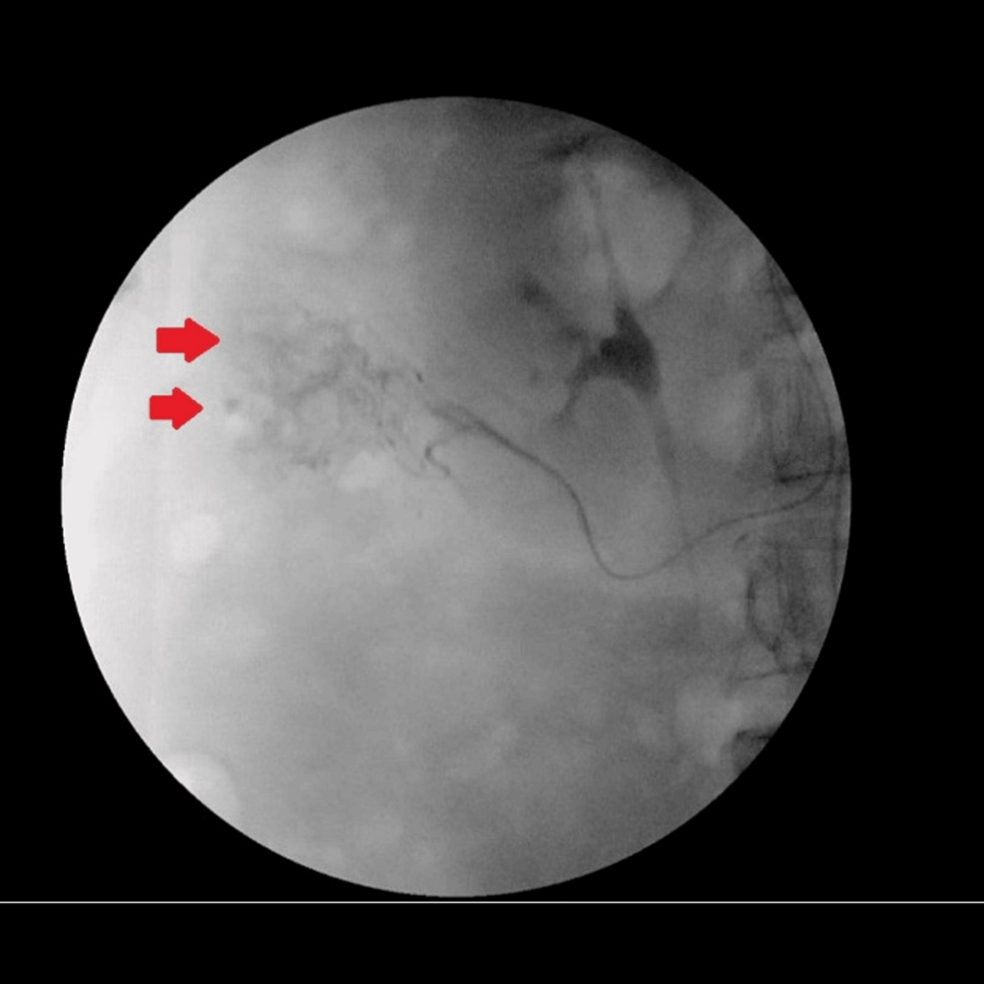
Super-selective ascending colon arteriogram showing bleeding from the ascending colon culprit artery (red arrows)

**Figure 3:**
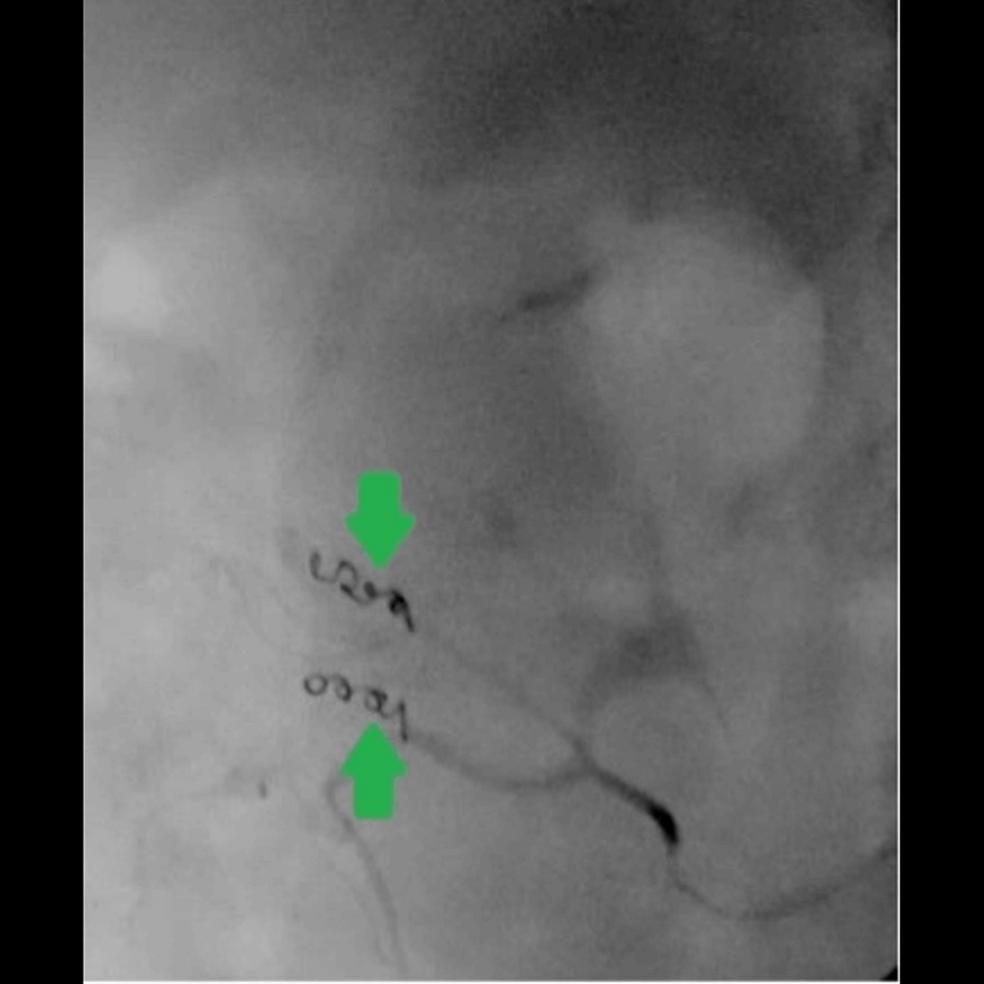
Post-embolization super-selective ascending colon angiogram showing no evidence of active bleeding after embolization with two coils (green arrows)

The interventional radiology (IR) team was consulted, and the patient underwent a conventional angiogram. They performed successful embolization of ascending colon arterial culprit territory causing diverticular and angiodysplastic bleeding using two coils (Interlock Boston Scientific), with abdominal aortogram, pelvic angiogram, and selective and super-selective visceral superior mesenteric arterial angiogram. This is shown in Figures 2-3.

The patient was transferred to the ICU for close monitoring. She did not have any further episodes of hematochezia after the embolization. The patient's clinical condition remained stable, and she was discharged from the hospital on the fourth day. The patient had no complications and was scheduled for outpatient follow-up appointments.

## Discussion

LGIB is known to be associated with poor outcomes. Estimates of the incidence of LGIB globally vary from 20.5 to 87.0 per 100,000 person-years [4]. In the United States, LGIB incidence varies from 20.5 to 41.8 per 100,000 person-years [4]. There are many causes of LGIB, including diverticulosis, vascular malformations, and cancer [5]. The clinical features of LGIB include hematochezia, melena, orthostasis, and hemorrhagic shock [1].

Diagnosis requires clinical and radiological evidence. An elevated blood urea nitrogen-to-creatinine ratio or the presence of blood clots usually suggests an upper GI source of bleeding [6]. Clinical investigations for LGIB are relatively non-specific. Therefore, imaging must be utilized for optimal and timely results.

CT angiography is increasingly being utilized for the diagnosis of acute GI bleeding. Considering the strength of adaptability of the technique, the study is useful in hemodynamically stable patients. Usually, CT angiography is performed as a three-phase scan beginning from a non-contrast image and ending with venous phase imaging [7]. Active extravasation of contrast on the arterial phase is one critical finding associated with active bleeding that has a bleeding threshold of 0.3-0.5 mL/min [7]. Additionally, the change of the jet color on venous imaging is pathognomonic of the condition [7]. Because of these properties, CT angiography can localize the source of bleeding, which can be followed by traditional angiography for treatment. The diagnostic accuracy of CT angiography has increased as higher spatial and temporal resolution are provided by multidetector CT technology [8,9]. A meta-analysis of nine studies published between 1995 and 2009 showed that angiography might fail to locate the source of GI bleeding despite a positive CT scan [8]. CT scan has a higher sensitivity in diagnosing GI bleeding than a colonoscopy. This is because it takes time to prepare the patient for a colonoscopy when the bleeding might have resolved [8,10].

A tagged RBC scan is generally used for GI bleeding in the mid and lower GI tracts. The diagnostic potential for an occult bleed is lesser. It is also used for risk stratification and appropriate intervention if needed [11]. As discussed previously, CT angiography significantly visualized active GI bleeding and its location compared to a tagged RBC scan [10]. However, using the two modalities did not impact the average hospital length of stay, blood transfusion requirement, the incidence of acute kidney injury, or in-hospital mortality [10].

Endoscopy remains the gold standard for the diagnosis of LGIB [1]. Studies revealed that the time for diagnosis of a bleed was significantly less for CT angiography compared to colonoscopy. Active bleeding was identified more accurately in CT angiography and colonoscopy [12]. However, the management of bleeding required surgery in patients with CT angiography compared to colonoscopy [12]. Therefore, for time-sensitive and initial screening results, CT angiography is one of the promising tools, as bowel preparation (which can take up to several hours) is not required. The treatment modality combined with traditional angiography is usually embolization [13]. Depending on the location of the bleeding, the approach is different. In the case of the superior mesenteric artery, the catheter is advanced to the vasa rectum, whereas for the inferior mesenteric artery, the catheter must be moved to the marginal or terminal artery [11]. IR embolization is being increasingly used in older patients, particularly because of the relatively less invasive nature of the procedure [14,15]. The complication associated with IR embolization includes bowel infarction owing to a lack of collateral supply. Other complications of the procedure include access-site hematomas (3-17%), pseudo-aneurysms, arterial dissection, ischemia (2.7%), and coil migration (3%) [15].

A large retrospective observational study done on patients with LGIB in more than 1,000 hospitals in 46 states in the United States showed an overall mortality rate of 0.6% [16]. Timely diagnosis of these diseases can aid in reducing mortalities and decreasing overall healthcare costs.

## Conclusions

CT angiography is an important tool in the timely management of LGIB, especially in elderly patients. Further large-scale studies are needed to investigate and confirm these findings.
